# Vasopressor Therapy and the Brain: Dark Side of the Moon

**DOI:** 10.3389/fmed.2019.00317

**Published:** 2020-01-10

**Authors:** Nicholas Heming, Aurélien Mazeraud, Eric Azabou, Pierre Moine, Djillali Annane

**Affiliations:** ^1^General Intensive Care Unit, Raymond Poincaré Hospital, Garches, France; ^2^U1173 Lab Inflammation and Infection, University of Versailles SQY-Paris Saclay - INSERM, Montigny-le-Bretonneux, France; ^3^Department of Neuro-Anesthesiology and Intensive Care Medicine, Sainte-Anne Teaching Hospital, Paris-Descartes University, Paris, France; ^4^Department of Physiology, Assistance Publique-Hôpitaux de Paris, Raymond-Poincaré Hospital, Garches, France

**Keywords:** sepsis associated encephalopathy, delirium, coma, sepsis, septic shock

## Abstract

Sepsis, a leading cause of morbidity and mortality, is caused by a deregulated host response to pathogens, and subsequent life-threatening organ dysfunctions. All major systems, including the cardiovascular, respiratory, renal, hepatic, hematological, and the neurological system may be affected by sepsis. Sepsis associated neurological dysfunction is triggered by multiple factors including neuro-inflammation, excitotoxicity, and ischemia. Ischemia results from reduced cerebral blood flow, caused by extreme variations of blood pressure, occlusion of cerebral vessels, or more subtle defects of the microcirculation. International guidelines comprehensively describe the initial hemodynamic management of sepsis, revolving around the normalization of systemic hemodynamics and of arterial lactate. By contrast, the management of sepsis patients suffering from brain dysfunction is poorly detailed, the only salient point being mentioned is that sedation and analgesia should be optimized. However, sepsis and the hemodynamic consequences thereof as well as vasopressors may have severe untoward neurological consequences. The current review describes the general neurological complications, as well as the consequences of vasopressor therapy on the brain and its circulation and addresses methods for cerebral monitoring during sepsis.

## Introduction

Sepsis is characterized by life-threatening organ dysfunction following non-homeostatic host response to an infection (1). Sepsis associated encephalopathy (SAE), a transient and potentially reversible brain dysfunction, occurs during the course of sepsis of an extra neurological source. SAE is both a frequent and serious complication (2). Indeed, in sepsis, acute neurological dysfunction occurs in up to 70% of cases (3, 4). Altered mental status is a risk factor of poor outcome for infected patients in the emergency room or in the ward (1, 5). Imaging studies of the brain in SAE are in most cases unremarkable. Mechanisms underlying SAE include neuro-inflammation, excitotoxicity, and ischemia. Ischemia occurs because of macrocirculatory and/or microcirculatory defects. Vasopressors are a cornerstone of the management of septic shock. However, vasoactive drugs may have deleterious consequences on cerebral perfusion. We herein review how sepsis, *per se*, may affect the brain, as well as the direct and indirect cerebral consequences of vasopressor therapy in sepsis.

## Sepsis and the Brain

Clinical features of SAE include sickness behavior, delirium and coma (6). Sickness behavior, the initial adaptive response to neuro-inflammation, results from the interaction of the inflammatory cytokines interleukin (IL)-1 alpha and IL-1 beta, tumor necrosis factor (TNF)-alpha and IL-6 on the brain. Sickness behavior associates apathy, asthenia, anorexia, and social withdrawal (7, 8). Delirium, characterized by fluctuating awareness and attention (9–11), presents as two distinctive entities, hyperactive delirium, which is easy to recognize but is fairly rare and hypoactive delirium which is frequent but may easily be overlooked (12). Delirium is detected at the bedside, using specific scales including the Confusion assessment method for the intensive care unit (CAM-ICU) or the Intensive Care Delirium Screening Checklist (ICDSC) (11, 13). Delirium is associated with prolonged mechanical ventilation, increased length of ICU stay and increased mortality (14). Medications, including Haloperidol, Ziprasidone, or Simvastatine all failed to reduce the duration of delirium in high quality randomized controlled trials (15, 16). The most severe form of neurological involvement in sepsis is coma which is linked to increased mortality and brainstem dysfunction (17–19). Neurological status is quantified using the Glasgow coma score or the FOUR score, which also assesses the brainstem function (20, 21). Sepsis survivors may suffer from long term neurological sequelae, including ICU-acquired paresis and cognitive impairment with subsequent functional disabilities and poor quality of life (22–24).

## Pathophysiology of Sepsis Associated Encephalopathy (Figure 1)

SAE results from several mechanisms, of which neuro-inflammation, ischemia, and excitotoxicity are the main (25).

**Figure 1:**
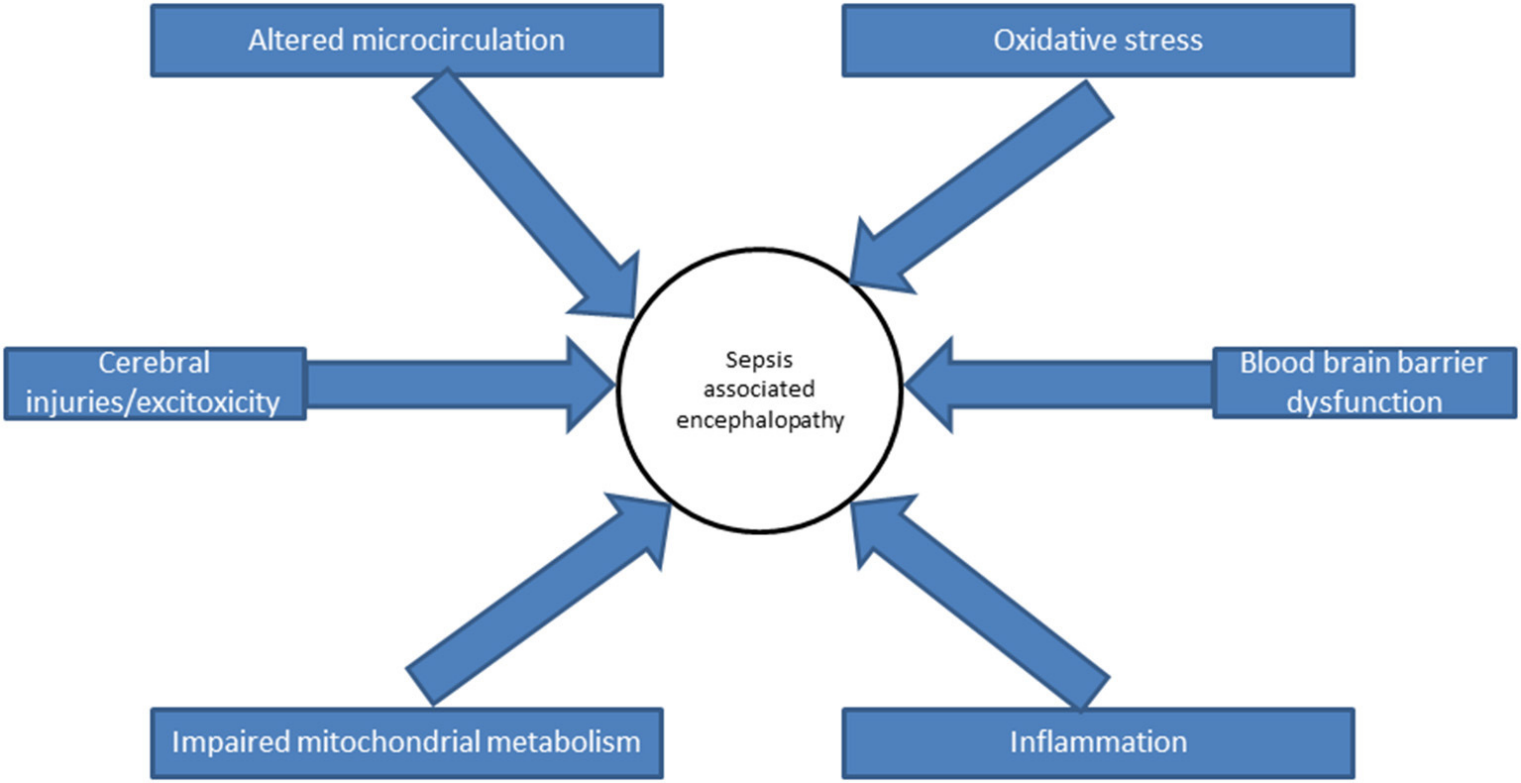
Pathophysiology of sepsis associated encephalopathy.

### Neuro-Inflammation

The blood-brain barrier is formed by endothelial cells with tight junctions, astrocyte endfeet and pericytes and isolates the cerebral tissue from potentially noxious circulating components. Circulating inflammatory components freely interact with cerebral tissue devoid of blood-brain barrier, the circumventricular organs (26, 27). Circulating cytokines may also be shuttled across the blood-brain barrier by specialized carrier proteins (28–30). Peripheral inflammation is sensed and transmitted by the vagal nerve to neurovegetative centers and the limbic system. Neuro-inflammation is subsequently mediated by microglial cells, the resident macrophages of the brain and by astrocytes, which support neuronal functions (31). Microglial cells express membrane-bound receptors that detect damage associated molecular patterns, and induce cellular activation. Microglial activation occurs early in experimental models of sepsis (32, 33) and is characterized by the production of pro inflammatory cytokines, such as tumor necrosis factor alpha, interleukin-1 beta and transforming growth factor beta (34). Statins administered to reduce inflammation did not lower the incidence of delirium in septic patient (35).

Low cerebral reserves of anti-oxidants make the brain particularly vulnerable to oxidative stress. Anti-oxidant reserves are depleted during sepsis (36). Inflammation in sepsis induces early oxidative stress (37), which may be responsible for subsequent cognitive impairment (38). Anti-oxidant drugs reduce neuroinflammation in experimental models of sepsis (39, 40).

### Ischemia

The adult human brain represents only 2% of the total body weight (41). Due to high metabolic demand, cerebral blood flow in healthy adults ranges from 750 to 900 ml/min, accounting for ~15% of an individual's resting cardiac output (42, 43). In physiological conditions, cerebral blood flow is modulated both at a macrocirculatory and microcirculatory level. Cerebral macrocirculation may be disrupted during sepsis, episodes of hypotension may alternate with hypertension leading to ischemic or hemorrhagic brain injuries (31, 44).

### Cerebral Macrocirculation

Adequate cerebral blood flow is critical for the proper function of the brain. Cerebral autoregulation refers to the capacity to maintain a constant cerebral blood flow, independently of systemic arterial pressure. In man, autoregulation occurs for mean arterial pressures between 60 and 150 mmHg (43). Beyond these values, cerebral blood flow becomes pressure-dependant and is therefore linearly correlated with cerebral perfusion pressure. Low mean arterial pressure leads to low cerebral blood flow. By contrast, excessive doses of vasoconstrictors may also lead to end-organ vasoconstriction and ischemia (45, 46).

Assessment of cerebral blood flow in septic patients is compounded by methodological difficulties. Most studies involve small populations and compare cerebral blood flow in sedated and ventilated septic patients to awake, non-septic control subjects (47, 48). A decrease in cerebral blood flow of the middle cerebral artery is consistently observed in experimental endotoxinemia (49, 50) and in sepsis (51–53). Such a decrease may be secondary to hyperventilation rather than the consequences of endotoxinemia/sepsis on cerebral hemodynamics. Sepsis also impairs cerebral autoregulation (54–57). Interestingly, decreased cerebral autoregulation in sepsis was found to be associated with delirium (58).

### Microcirculation

Cerebral energetic requirements relate to the functioning of neurons, rather than that of glial supporting tissue. Indeed, the generation of neuronal action potentials, through the active transmembrane transport of ions, requires large quantities of energy. Cerebral blood flow is inhomogeneous, increasing in areas where neuronal activity is the greatest (59). The metabolic rate is greater in the gray matter of the brain, where most the cell bodies lie, than in the white matter (60). Adequate cerebral blood flow at the cellular level is obtained through a functioning gliovascular unit, associating endothelial cells, astrocytes and pericytes (61). Microcirculatory cerebral blood flow adaptation is modulated by hydrogen ion concentration, partial carbon dioxide pressure, partial oxygen pressure as well as neurotransmitter concentration and intracellular calcium concentration (62, 63). Increased carbon dioxide or hydrogen ion concentrations or hypoxia lead to cerebral vascular vasodilation and greater cerebral blood flow (64).

Sepsis, by injuring endothelial cells and inducing the production of NO disrupts the blood brain barrier, allowing leucocytes and inflammatory cytokines to penetrate the brain, which in turn leads to neuroinflammation, thereby promoting brain dysfunction (65–67). Ischemic or hemorrhagic lesions in the brain may occur in the presence of disseminated intravascular coagulopathy, affecting up to one critically ill patient out of five (68). In addition, sepsis is associated with mitochondrial dysfunction, leaving neurons unable to properly use oxygen (69, 70).

The association of macro and microcirculatory dysfunction compounded by an incapacity to respond to metabolic needs, contribute to the formation of cerebral ischemic lesions (24). Indeed, post mortem studies of the brain of septic patients found evidence of ischemic lesions (44), which may in part explain the high prevalence of disability in sepsis survivors (24). Another well-documented risk factor for ischemic stroke is atrial fibrillation (71). Large retrospective studies report an increased risk of new onset atrial fibrillation during sepsis. In a cohort of more than 60,000 septic patients, atrial fibrillation occurred during 25.5% of hospitalizations (72). Prospective cohorts in the ICU confirmed the high incidence of new onset atrial fibrillation (73, 74). However, the exact prevalence of atrial fibrillation, which may be transient, is probably underestimated. Ischemic stroke is a major complication of atrial fibrillation (75). Large database studies report an increased risk of ischemic stroke associated with sepsis (75). Additionally, sepsis survivors having suffered from new onset atrial fibrillation exhibit a higher risk of subsequent stroke (76). Pathogens such as *Mycoplasma pneumoniae* are associated with an increased risk of stroke, possibly through immune mediated mechanisms (77). Other pathogens, including the varicella zoster virus, *Treponema pallidum* and *Streptococcus pneumoniae* may cause vasculopathy or vasculitis (78), while intracranial aneurysms or blebs, caused by an infection of the arterial wall are typically associated with *Staphylococcus aureus* or *Streptococcus* species endocarditis (79).

### Excitotoxicity

During sepsis, neuronal and microglial apoptosis occur mainly in the amygdala, *nucleus tractus solitarii* and *locus coeruleus* (44). Excitotoxic neuronal apoptosis is mediated by glutamate, which is produced in large quantities by activated microglial cells (80). Cerebrospinal fluid glutamate concentration correlates with the neurological state during bacterial meningitis (81). The adjunction of glutamate-rich cerebrospinal fluid to neuronal cell culture induces dose dependent cellular toxicity, which is attenuated by the adjunction of a NMDA receptor antagonist (82). Hydrogen sulfide and low doses of carbon monoxide also exhibit protective effects against glutamate-mediated neurotoxicity. Mitochondrial-mediated apoptosis occurs during sepsis, mediated by cellular pro-apoptotic factors (83, 84). Other pro-apoptotic factors, include, nitric oxide, TNFα, and hyperglycemia (85).

### Secondary Neurological Injuries Occurring During Sepsis

Any organ dysfunction occurring during sepsis may affect the proper functioning of the brain. These include but are not limited to, circulatory or cerebral auto-regulation impairment, systemic organ (hepatic, renal, metabolic, or respiratory) failure as well as the direct or indirect consequences of medication side-effects (opioids, sedatives, antibiotics, sodium disorders…) and environmental factors (rest or lack thereof, light, and noise exposure) (6).

## Cerebral Monitoring During Sepsis

Cerebral function assessment in sepsis is frequently overlooked. For instance, hypoactive delirium, while common, is underestimated. Means to accurately monitor the cerebral function at the bedside are not wildly available. Concomitant sedation may depress the brain function. No evidence or recommendation supports monitoring cerebral perfusion or function in septic patients (86). While the dose of vasopressors should be tailored to meet specific targets or surrogates of organ function, no guideline proposes neurological endpoints in sepsis. Nevertheless, several methods enable physicians to assess the cerebral function or perfusion. Methods used to assess cerebral function or perfusion include but are not limited to:

- Clinical Scores
  The simplest mean of monitoring the brain in an awake patient is clinical. Acute brain dysfunction is identified using validated scales for delirium (i.e., ICSDC or CAM-ICU), coma (Glasgow Coma Scale) or brainstem reflexes in comatose patients (FOUR score) (10, 11, 20, 21). Vasopressors are rarely, if ever, titrated to clinical surrogates of brain dysfunction (87). Preliminary data seem to indicate that during sepsis mean arterial blood pressure of 80–85 mmHg rather than 65–70 mmHg may mitigate brain dysfunction (88).
- Biomarkers
  Several biomarkers have been promoted to diagnose or manage brain injuries; including brain injuries of a septic origin. Elevated levels of protein S100B, neuron-specific enolase (NSE) or neurofilament have been reported during SAE. However, their use is controversial since extra-neurological tissues may also release these proteins (89–92).
- Neuroimaging
  Cerebral blood flow may be noninvasively monitored by transcranial Doppler ultrasound at the bedside. Blood flow velocity in the cerebral mean artery, a surrogate for cerebral blood flow, is measured using sound waves. No impact of transcranial Doppler ultrasound use on patient centered outcomes has ever been demonstrated. Additionally, inadequate acoustic windows for transcranial doppler monitoring may occur in up to 10% of patients (93).
  Neuroimaging, using computed tomography or magnetic resonance imaging of the brain may help demonstrate structural injury to the central nervous system. Imaging studies in septic patients with neurological involvement found evidence of white matter hyperdensities and of ischemic stroke (94–96). Such anomalies may be associated with long term cognitive impairment (24). Drawbacks of imaging studies include: impractical for continuous monitoring, do not accurately predict the functional state of the patient; and the most recent technology might not be available in every hospital.
  Dynamic methods, including 18F-fluorodeoxyglucose (FDG) PET imaging and functional MRI go beyond a simple exploration of cerebral morphology by exploring cerebral activity. Dynamic neuroimaging techniques, while not routinely used, may be helpful in predicting long term outcomes in critically ill patients (97–99).
- Electroencephalogram
  The electroencephalogram (EEG) records the neuronal electrical activity at the surface of the scalp; indirectly informing on the quality of cerebral perfusion. The EEG is non-invasive and easily available at the bedside (100). EEG patterns may be modified during sepsis. Continuous generalized triphasic waves and burst suppression are associated with the severity of brain dysfunction and with mortality (101). Delta-predominant background, absence of EEG reactivity, periodic discharges are independently associated with mortality (102, 103). However, none of these patterns are specific of sepsis.
- Evoked Potentials
  Sensory evoked potentials are generated in response to somatosensory, visual or auditory stimuli. Evoked potentials may be obtained non-invasively at the bedside (100). Sensory evoked potentials explore the integrity of the peripheral or cranial nerve, the spinal cord and/or the brainstem, the thalamus and the cortex. Septic encephalopathy is associated with impaired somatosensory evoked potentials (104, 105). Prolonged nervous conduction times hint at an acute brain dysfunction and are prognostic markers in the critically ill (106, 107).
- Intracranial Pressure
  The ideal mean of estimating brain perfusion at the bedside is through the assessment of cerebral perfusion pressure. Since the brain is enclosed in a rigid cranium, cerebral perfusion pressure (CPP) is related to mean arterial pressure (MAP) and intracranial pressure (ICP) by the equation CPP = MAP—ICP. Brain injury leading to elevated ICP will reduce CPP if blood pressure remains identical. During severe brain injuries, vasopressors will maintain MAP but may also induce extreme vasoconstriction in the injured zones of the brain, lowering blood flow in these regions, thereby potentially worsening cerebral injuries (108). Optimal blood pressure strikes a delicate balance between transcapillary hydrostatic and oncotic forces and acceptable cerebral perfusion pressure (108). Only one study in sepsis assessed ICP without finding any evidence of intracranial hypertension (109). Intracranial pressure is almost never directly measured in sepsis, even in severe central nervous system infections, which are theoretically the most at risk of intracranial hypertension. Routine monitoring of intracranial pressure is not recommended in sepsis (110, 111).
- Cerebral Oximetry
  Near-infrared spectroscopy uses the principle of light transmission and absorption to determine the tissue concentration of oxyhemoglobin and deoxyhemoglobin and to calculate tissue oxygen saturation. Cerebral oxygen saturation is measured at the frontal lobe and is used as a surrogate for cerebral blood flow. Decreased cerebral oxygen saturation during sepsis may be associated with an increased risk of death (112). Cerebral tissues oxygenation indexes assess cerebral autoregulation in septic patients (56). The exact role of cerebral oximetry for monitoring the cerebral function in sepsis needs to be defined (113).

## Effect of Vasopressors on the Brain

### Direct Effect

Moderate doses of norepinephrine increase cerebral vascular resistances and moderately decrease cerebral blood flow in isolated perfused dog brains (114). In healthy volunteers, norepinephrine lowers cerebral blood flow by increasing cerebral vascular resistances (115). The systemic administration of low doses of dopamine or norepinephrine in healthy piglets increases cerebral oxygenation (116, 117). High doses of norepinephrine administered to healthy rodents induce heterogeneous increases of cerebral blood flow and disruption of the blood brain barrier (118). The infusion of high doses of norepinephrine in healthy volunteers negatively affects cerebral oxygenation (45). The adjunctive administration of vasopressin in sepsis did not alter the number of days alive without neurological dysfunction (119). The systemic administration of moderate doses of angiotensin II to healthy pigs increases carotid blood flow; the effect on cerebral blood flow was not reported (120). The systemic administration of high doses of angiotensin to healthy baboons lead to disruption of the blood brain barrier and to ischemic brain lesions (121). In healthy humans, the intracarotid administration of angiotensin did not change regional cerebral blood flow (122, 123).

### Indirect Effect

New onset atrial fibrillation in the ICU is linked to the presence of endogenous or exogenous vasopressors. A randomized trial comparing the administration of norepinephrine plus dobutamine vs. epinephrine in the treatment of sepsis found that overall 2% of the population developed an ischemic stroke, and 1% of the population developed cerebral bleeding over the first 3 months (124). Both the incidence of supraventricular arrhythmia and of stroke was similar in patients treated by norepinephrine plus dobutamine vs. epinephrine (124). The incidence of cardiac arrhythmia is greater with dopamine than with norepinephrine (125, 126). The adjunctive administration of vasopressin in sepsis did not alter the prevalence of cerebrovascular accidents (119). The administration of angiotensin II in vasodilatory shock is not associated with an increased risk of brain injury (127).

Little data is available regarding goals for neuroprotection during sepsis. Higher blood pressure targets may be associated with mortality (128). Current guidelines indicate that the optimal MAP target to reduce mortality during sepsis is 65 mmHg (86). MAP target personalization remains to be formally evaluated.

## Conclusions

Neurological dysfunction is frequent during sepsis. Both sepsis and high dose vasopressor therapy may negatively impact cerebral perfusion and/or oxygenation. The best way to monitor and to manage patients suffering from sepsis-induced neurological dysfunction remains to be elucidated.

## Author Contributions

NH conceived the manuscript. NH, AM, EA, PM, and DA contributed to the literature search and wrote the manuscript.

### Conflict of Interest

The authors declare that the research was conducted in the absence of any commercial or financial relationships that could be construed as a potential conflict of interest.
